# Beta 1,3-1,6 Glucans Produced by Two Novel Strains of Aureobasidium Pullulans Exert Immune and Metabolic Beneficial Effects in Healthy Middle-aged Japanese Men: Results of an Exploratory Randomized Control Study

**DOI:** 10.14283/jarlife.2023.11

**Published:** 2023-07-28

**Authors:** N. Ikewaki, T. Sonoda, G. Kurosawa, M. Iwasaki, V. Devaprasad Dedeepiya, R. Senthilkumar, S. Preethy, S.J.K. Abraham

**Affiliations:** 1 Dept. of Medical Life Science, Kyushu University of Health and Welfare, Japan; 2 Institute of Immunology, Junsei Educational Institute, Nobeoka, Miyazaki, Japan; 3 Department of Academic Research Support Promotion Facility, Center for Research Promotion and Support, Fujita Health University, Aichi, Japan; 4 MabGenesis KK, Nagoya, Japan; 5 Centre for Advancing Clinical Research (CACR), University of Yamanashi - School of Medicine, Chuo, Japan; 6 Mary-Yoshio Translational Hexagon (MYTH), Nichi-In Centre for Regenerative Medicine (NCRM), Chennai, India; 7 Fujio-Eiji Academic Terrain (FEAT), Nichi-In Centre for Regenerative Medicine (NCRM), Chennai, India; 8 Antony- Xavier Interdisciplinary Scholastics (AXIS), GN Corporation Co. Ltd., Kofu, Japan; 9 R & D, Sophy Inc., Japan; 10 Levy-Jurgen Transdisciplinary Exploratory (LJTE), Global Niche Corp, Wilmington, DE, USA

**Keywords:** AFO-202, N-163 strains of black yeast, Aureobasidium pullulans, beta glucans, immune enhancement, immunomodulation, glucotoxicity, lipotoxicity, metabolism, COVID-19, fatty liver disease

## Abstract

**Objectives:**

In this pilot study, we have evaluated the specific metabolic and immune-related benefits of the AFO-202 strain and N-163 strain of black yeast Aureobasidium pullulans-produced beta 1,3-1,6 glucan in healthy human subjects.

**Methods:**

Sixteen healthy Japanese male volunteers (aged 40 to 60 years) took part in this clinical trial. They were divided into four groups (n = 4 each): Group I consumed AFO-202 beta-glucan (2 sachets of 1 g each per day), IA for 35 days and IB for 21 days; Group II consumed a combination of AFO-202 beta-glucan (2 sachets of 1 g each) and N-163 beta-glucan (1 sachet of 15 g gel each per day), IIA for 35 days and IIB for 21 days.

**Results:**

Decrease in HbA1C and glycated albumin (GA), significant increase of eosinophils and monocytes and marginal decrease in D-dimer levels, decrease in neutrophil-to-lymphocyte ratio (NLR), with an increase in the lymphocyte-to-CRP ratio (LCR) and leukocyte-to-CRP ratio (LeCR) was observed in Group I between pre- and post-treatment. Decrease in total and LDL cholesterol, a decrease of CD11b, serum ferritin, galectin-3 and fibrinogen were profound in Group II between pre- and post-treatment. However, there was no statistically significant difference between day 21 and day 35 among the groups.

**Conclusion:**

This outcome warrants larger clinical trials to explore the potentials of these safe food supplements in the prevention and prophylaxis of diseases due to dysregulated metabolism, such as fatty liver disease, and infections such as COVID-19 in which balanced immunomodulation are of utmost importance, besides their administration as an adjunct to existing therapeutic approaches of both communicable and non-communicable diseases.

**Figure:**
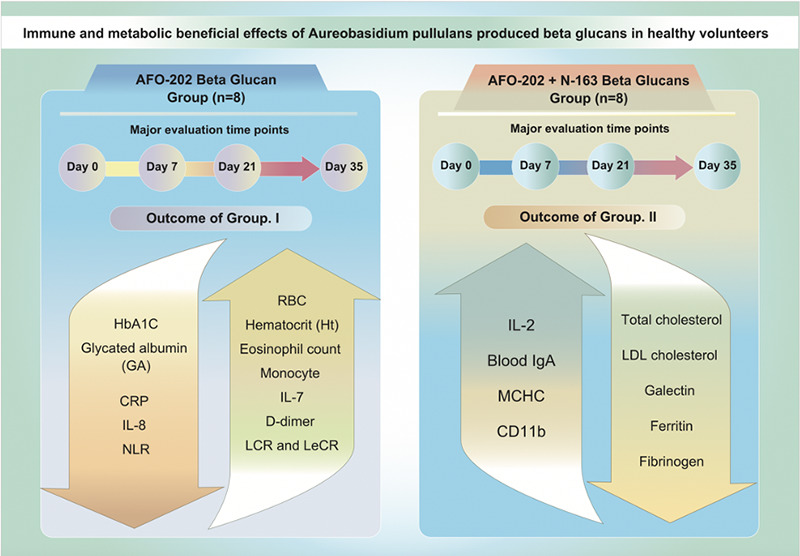
Graphical Abstract

## Introduction

**M**etabolism imbalance is a gradually occurring condition leading to diabetes, heart disease, stroke, etc., and the risk varies between populations based on their genetic predisposition, diet, lifestyle, and environmental influences (1). When an individual is diagnosed with any lifestyle illness requiring medication, further prevention and deceleration of the pathogenesis is an uphill task. To address this, exercise, dietary modifications such as intake of foods with low glycaemic index or fats, and medications are advised which are temporary and not definitive solutions (1). This study aims to study the effects of Aureobasidium pullulans AFO-202 and N-163 strains-produced biological response modifier beta glucan food supplements in middle-aged, healthy subjects. The rationale behind this objective is explained below.

The liver, being the metabolic centre of the body, often becomes the key target (2), and coronary artery and cerebral systemic coagulopathies (1) may also occur. Additionally, the immune reserves may be depleted in handling, and the circulating high levels of advanced glycation end products (AGEs) and lipids affect the functional capability of the immune cells, leading to high risk of disease severity (3) when COVID-19-like infections occur (4). The fibrosis that ensues after a chronic inflammation-metabolic-immune dysregulation can lead to pulmonary or liver fibrosis, such as non-alcoholic steatohepatitis (NASH) (2), which could eventually culminate in carcinogenesis (5). Glucotoxicity and lipotoxicity also cause gut dysbiosis (6), which is now increasingly considered the key factor influencing the progression of infections, inflammations, and fibrosis, creating a vicious cycle.

Against the given background, as a remedy, what we require is an agent which should be safe and possesses the following potentials.

### Regarding glucotoxicity and lipotoxicity

#### At an early stage or before onset of disease

It should be able to balance the blood glucose levels, especially the post-prandial spike and balance the blood cholesterol level without any side effects.

#### Post-disease onset stage

During and after onset of the glucotoxicity and/ or lipotoxicity, it should be able to control abnormal glucose and cholesterol levels without any side effects and without any adverse interactions with other drugs prescribed. It should be able to beneficially regulate LDL and VLDL without adversely affecting HDL and should be able to control inflammation and the accumulation of free fatty acids (FFA).

#### After progression of disease with chronic sequalae stage

It should be able to control organ inflammatory reaction to avoid fibrosis and also balance micro-inflammation of the gut.

***Regarding systemic wellness and immune balance, throughout the various stage, mentioned above, it should support*** the immune system, especially during aging, by enhancing it to prevent illnesses from disease-affected weakness. It should be able to promote immune modulation to avoid hyper-activation and cytokine storm. It should have potential to balance immune enhancement and modulation to avoid pre-disposing factors to carcinogenesis and also reverse gut dysbiosis.

Although a single such prophylactic measure or component is almost impossible, we selected two products of strains from the black yeast A. pullulans which have a track record of safety (7-9) and potential to restore the gut microbiome (10, 11).

##### AFO-202 benefits

The AFO-202 strain-produced beta glucan has been shown to normalize Hba1c and fasting, post-prandial blood glucose levels in patients with type II diabetes (7). It has been shown to decrease elevated LDL and VLDL cholesterol and triglycerides in clinical studies of metabolic syndrome (8). Enhancement of immune cells such as natural killer (NK) cells and macrophages, apart from suppression of pro-inflammatory cytokines while enhancing beneficial cytokines and antibodies has been reported (9). Apart from these beneficial immune and metabolic modulations, a decrease in the neutrophil-to-lymphocyte ratio (NLR) and increase in lymphocyte-to-C-reactive protein (CRP) ratio (LCR) and leukocyte-to-CRP ratio (LeCR) are particularly significant in COVID-19 (12), as the dysregulation of these parameters has been correlated with progression of the disease and higher odds of mortality (13).

##### N-163 benefits

While AFO-202 is relevant to both metabolic and immune regulation, the anti-inflammatory, anti-fibrotic potential of N-163 has been reported with significance in a NASH animal model (14), along with a decrease in inflammation-associated lipid parameters such as non-esterified free fatty acids (NEFAs) (15). Thus, N-163 is more relevant in the stages of progressed disease status.

The potential of the AFO-202 and N-163 beta glucan as an immune adjuvant in the prophylaxis of COVID-19, along with beneficial anti-coagulopathy benefits in clinical trials, has been described (16-20). These beta glucans have also been effective as inti-infective agents against viral infections such as dengue, influenza, rabies apart from beneficial immune-modulation in sepsis (21-24).

Before addressing specific disease targets, we sought to study the effects of AFO-202 and N-163-produced beta glucans in the middle-aged, healthy subjects, as they have been the most vulnerable population for metabolic diseases (25) and severe COVID-19.

## Methods

The study was conducted in compliance with the ethical principles based on the Declaration of Helsinki. The study protocol was approved by the institutional review board (IRB) of Chiyoda Paramedical Care Clinic, Tokyo, Japan (study protocol number GNC20C1), and registered with the University Hospital Medical Information Network-Clinical Trial Registry (UMIN-CTR) of Japan, Trial registration Number UMIN: 000040882 (26). The study was conducted at the Chiyoda Paramedical Care Clinic, Tokyo, Japan.

### Patient and Public involvement

The subjects and the public were involved in the design and conduct of this research. During the feasibility stage, priority of the research question, choice of outcome measures, and methods of recruitment were informed by discussions with the subjects through a focus group session and structured interviews. Once the trial is published, participants will be informed of the results through a study newsletter suitable for a non-specialist audience.

### Study Subjects

The study was designed as an exploratory study in sixteen healthy Japanese male volunteers aged 40 to 60 years with four intervention conditions: two test food groups and two durations of intake in each test food group.

The person in charge of the allocation, as specified in the study protocol, allocated the study subjects to the four groups as evenly as possible, giving first priority to pretest BMI, second priority to weight, and third priority to height.

Subjects who met the selection criteria (26) and did not fall under any of the exclusion criteria were eligible for the study.

The CONSORT flow diagram of the study is available in the supplementary material.

### Intervention

The duration of the study food intake and the schedule of visits for each group was:

### Group I

AFO-202 beta glucan (1g containing 42 mg active ingredient) – 2 sachets with each meal

- IA: Intervention for 35 days
- IB: Intervention for 21 days

### Group II

AFO-202 beta glucan (1g containing 42 mg active ingredient) – 2 sachets with each meal + N-163 (15 g gel sachet containing 90 mg of the active ingredient) – 1 sachet with any one of the meals

- IIA: Intervention for 35 days
- IIB: Intervention for 21 days

### Primary endpoints

1. Immune activation effect
2. WBC, RBC, Hb, Ht, PLT, MCV, MCH, MCHC
3. Basophils, eosinophils, neutrophils, lymphocytes, monocyte counts
4. CRP, IgG in blood, IgM in blood, IgA in blood
5. IL-2, IL-6, IL-7, IL-8, IFN-γ, sFas ligand

### Secondary endpoints

1. Coagulopathy related markers
  - Ferritin, D-dimer, PT, Fib, CD11b in monocyte fraction, galectin-3
2. Blood glucose level
3. HbA1c, GA
4. Cholesterol level
  - TG, T-Cho, HDL-Cho, LDL-Cho

### Safety evaluation items

Incidence of adverse effects.

### Evaluations

#### At pre-test and before intake

Blood sampling volume: 34 mL

Background survey was performed to gather information on the gender, date of birth, age, smoking habits, drinking habits, eating habits, current medical history, medication, treatment, previous history, allergies (to drugs and food), regular use of food for specified health uses, functional foods, health foods, intake of foods rich in β-glucan foods containing beta-glucan, intake of immunity-boosting foods, and blood donation (within 1 year).

The following assessments were performed,

- Medical history and physical measurements: medical history, height, weight, BMI, temperature
- Physiological examination: systolic blood pressure, diastolic blood pressure, pulse rate
- Haematology, cellular immunology assessments and blood biochemistry

#### Day 21 of intake and Day 35 of intake

- Blood collection volume: 31 mL
- History and physical examination: history, weight, BMI
- Physiological examination: systolic blood pressure, diastolic blood pressure, pulse rate
- Haematology, cellular immunology assessments and blood biochemistry

#### Daily diary

The diary was maintained from the day of the start of the consumption of the test food until the 35th day of consumption. The following items were recorded in the diary.

Intake of test foods, body temperature, intake of food for specified health uses, functional foods, and health foods, intake of restricted foods, subjective symptoms, visits to medical institutions, treatment, and use of medicines.

#### Examples of restricted foods

Supplements rich in beta-glucan: supplements containing beta-glucan extracted and concentrated from yeast, barley, mushrooms and seaweed.

Foods claiming to stimulate the immune system: yoghurt, lactobacillus beverages, bifidobacteria powder, propolis, lactoferrin, etc.

### Statistical analysis

The statistical significance level was set at 5%, two-sided. SPSS26.0 (IBM Japan, Ltd.) and Microsoft Excel (Microsoft Corporation) were used as analysis software. An unpaired t-test, Fisher’s exact test (Bonferroni correction), Dunnett certification, and a correspondence t-test were performed.

## Results

One study subject (No. 4) with leukocyte abnormalities (suspected leukaemia) discontinued or dropped out of the study. In addition, two study subjects (Nos. 11 and 16) were excluded because they fell under “6) Other obvious reasons for omission” in the “Exclusion criteria for PPS analysis” (26) section. After excluding these two subjects from the FAS, 13 subjects were included in the PPS.

Comparisons between the test food groups using the change from pre-consumption values showed statistically significant differences in the parameters outlined in Table 1.

**Table 1.** AFO-202 vs AFO-202+N-163 Beta glucans in healthy volunteers; results in a nutshell

Beneficial effect	Biomarker	Outcome	Effective compound	P Value significance
Immune enhancement	Monocyte	Increase	AFO-202	<0.05
	Eosinophils		AFO-202	<0.05
	CRP		AFO-202	
	IL-7		AFO-202	<0.05
	IL8		AFO-202	<0.05
Immmunomodulation	IgA	Increase	AFO-202 +N-163	<0.01
	IL-2		AFO-202 +N-163	<0.05
	LCR		AFO-202 +N-163	
	LeCR		AFO-202 +N-163	
	NLR	Decrease	AFO-202 +N-163	<0.05
Metabolic regulation	HbA1c	Decrease	AFO-202	<0.05
	Glycated Albumin		AFO-202	<0.05
	T-Cho		AFO-202 +N-163	<0.05
	LDL-Cho		AFO-202 +N-163	<0.01
Coagulopathy prevention	D-Dimer	Decrease	AFO-202	<0.05
	Ferritin		AFO-202 +N-163	
	Fibrinogen		AFO-202 +N-163	
	Galectin-3		AFO-202 +N-163	
	CD11b	Increase	AFO-202 +N-163	

Abbreviations: CRP- C-reactive protein; IL-Interleukin; Ig-Immunoglobin; LCR-Lymphocyte to C-reactive protein ratio; NLR- Neutrophil to Lymphocyte ratio; LeCR-Leukocyte to C-reactive protein ratio; LDL-Low density lipoprotein

### AFO-202 beta glucan

#### Glucose metabolism

##### HbA1C

**Figure 1.**
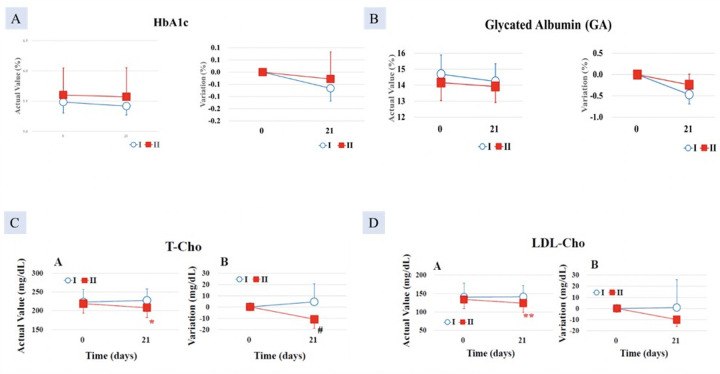
Decrease in A. HbA1c; B. Glycated albumin (GA); significantly greater in Group I (AFO-202 beta glucan) compared to Group II (AFO-202+N-163 beta glucan); Decrease in C. total cholesterol (T-Cho) and; D. LDL-cholesterol significantly greater in Group II (AFO-202+N-163 beta glucan) compared to Group I (AFO-202 beta glucan)

In Group I, the decrease post-intervention was greater by -0.23 ± 0.06% after 35 days of intake compared with Group II (-0.08 ± 0.05%), which showed a statistically significant higher value (p < 0.05) (Figure 1A).

##### Glycated albumin (GA)

After 21 days of consumption, the GA decrease in Group I (-0.53 ± 0.15%) was statistically significantly higher than that of the Group II (-0.10 ± 0.18%) (p < 0.05), (Figure 1B).

#### Haematological indices of immune stimulation

##### RBC

After 21 days of consumption, the RBC was statistically significantly higher (p < 0.05) in Group I (4.0 ± 5.3 x 104/ μL) compared with test Group II (-8.8 ±5.6 x 104/μL).

##### Hb

After 21 days of consumption, the value in Group I (0.13 ± 0.12 g/dL) (p < 0.01) was statistically significantly higher compared with that of Group II (-0.38 ± 0.15 g/dL). Haematocrit (Ht)

After 21 days of intake, Group I (-0.03 ± 0.40%) showed statistically significant higher Ht values than did Group II (-1.50 ± 0.29%) (p < 0.01).

##### Eosinophils

**Figure 2.**
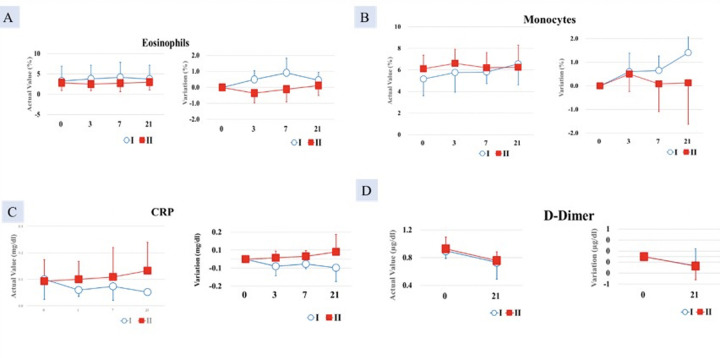
Increase in A. Eosinophil count; B. Monocytes count significantly greater in Group I (AFO-202 beta glucan) compared to Group II (AFO-202+N-163 beta glucan); decrease in C. CRP and D. D-Dimer, significantly greater in Group I (AFO-202 beta glucan) compared to Group II (AFO-202+N-163 beta glucan)

A statistically significant difference was found between the test food groups in terms of the change from pretreatment to post-treatment (p < 0.05). Eosinophil count (0.50 ± 0.54%) was higher in Group I compared with Group II (-0.36 ± 0.61%) (Figure 2A).

##### Monocytes

After 7 days of consumption, Group I (6.63 ± 0.51%) showed a statistically significantly higher monocyte value than did Group II (5.00 ± 0.82%) (p < 0.05). After 21 days of consumption, Group I (1.93 ± 0.47%) also showed a statistically significant increase compared with Group 2 (0.87 ± 0.21%) (p < 0.05) (Figure 2B).

##### CRP

At 21 days, the decrease in CRP was greater in Group I (level= 0.0517 mg/dl) compared with Group II (0.1329 mg/dl), which was statistically significant (p < 0.05) (Figure 2C).

##### IL-7

After 7 days of consumption, the IL-7 level was statistically significantly higher (p < 0.05) in Group I (4.33 ±0.87 pg/mL) compared with Group II (2.67 ±0.55 pg/mL).

##### IL-8

Group I’s IL-8 values (7.003 ±0.929 pg/mL) were statistically significantly higher than those of Group II (5.230 ±0.469 pg/mL) after 7 days of intake (p < 0.05).

##### D-dimer

After 35 days of intake, the D-dimer decrease in Group I (-0.30 ±0.10 μg/mL) was statistically significantly higher than that of the test food Group II (0.00 ±0.10 μg/mL) (p < 0.05) (Figure 2D).

##### NLR, LCR, and LeCR

**Figure 3.**
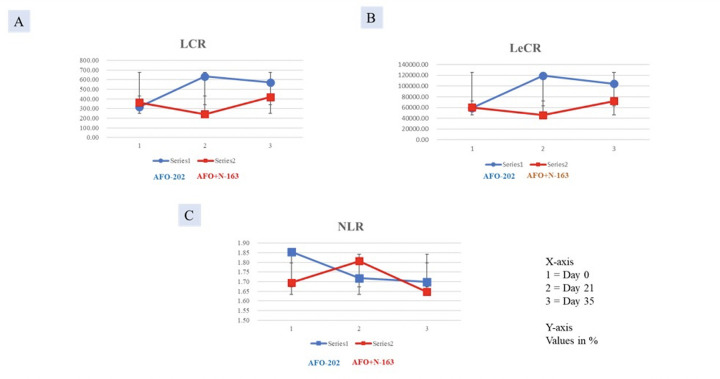
Increase in A.LCR; B. LeCR and decrease in C. NLR significantly greater in Group I (AFO-202 beta glucan) compared to Group II (AFO-202+N-163 beta glucan)

The decrease in NLR was greater in Group I at day 21, but at day 35, the decrease was higher in Group II. In terms of LCR and LeCR, at day 35, the increase from baseline value was greater in Group I compared with Group II (Figure 3A-C). The results however were not statistically significant.

### N-163

#### Regulation of lipid parameters

##### Total cholesterol (T-Cho)

After 21 days of intake, the T-Cho decrease in Group II (-12.8 ± 4.0 mg/dL) was statistically significantly higher than that of the test food Group I (9.0 ± 12.3 mg/dL) (p < 0.05) (Figure 1C).

##### LDL cholesterol (LDL-Cho)

There was a statistically significant decrease in LDL-Cho in Group II, at 124.0 ±25.3 mg/dL, after 21 days of consumption, compared with 134.0 ±25.2 mg/dL before consumption (p < 0.01) (Figure 1D).

#### Immuno-modulation and anti-inflammatory effects

##### IL-2

The increase to 0.3743 ± 0.1165 pg/mL after 14 days of post-observation in Group II was statistically significant higher (p < 0.05) than the 0.1220 ± 0.0635 pg/mL value in Group 1.

##### Blood IgA

After 21 days of intake, Group II (340.3 ±64.9 mg/dL) had a statistically significantly higher blood IgA value than did Group I (175.0 ±9.5 mg/dL) (p < 0.01) (Table 1).

##### MCHC

After 7 days of consumption, the MCHC was statistically significantly higher (p < 0.05) in Group II (32.56 ± 0.55%) compared with Group I (31.85 ± 0.55%).

##### Serum galectin, ferritin, and fibrinogen

**Figure 4.**
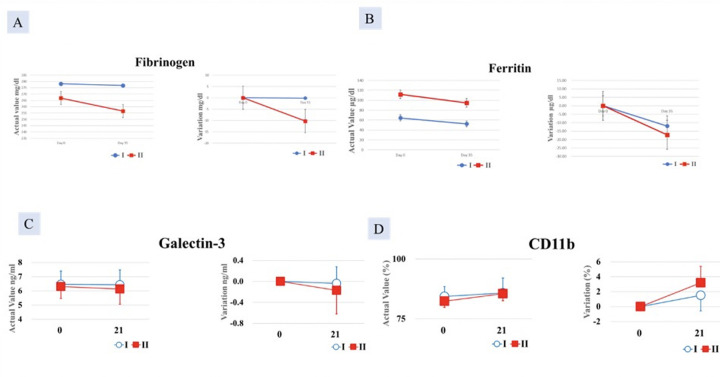
Decrease in A. Fibrinogen and; B. Ferritin C. Galectin-3 and D. increase in CD11b greater in Group II (AFO- 202 + N-163 beta glucan) compared to Group I (AFO-202 beta glucan)

The decrease in serum fibrinogen, ferritin and galectin-3 was greater in Group II compared with Group I, but the difference was not significant (Figure 4A-C).

##### CD11b

An increase in CD11b in the monocyte fraction was observed in Group II after 21 days of ingestion compared with Group I but it was not statistically significant (Figure 4D).

##### Other parameters

No statistically significant difference was observed in the other parameters. There was no discernible difference in the food consumption practices in the individuals, post-intervention.

##### Safety endpoints (incidence of adverse reactions)

No adverse reactions occurred in this study.

## Discussion

The results of the study has proven the hypothesis suggest that A.pullulans produced beta glucans exert beneficial metabolic and immune effects, with the AFO-202 beta glucan capable of eliciting beneficial effects in balancing blood glucose, alleviating glucotoxicity with immune activation, while a combination of AFO-202 and N-163 beta glucans has significant anti-inflammatory and lipid profile regulating potential, thereby alleviating lipotoxicity.

Metabolic syndrome (MeTS) is a significant health issue in today’s world, affecting one quarter of the global population, which amounts to over a billion people (27). Although lifestyle changes remain the primary modality of therapy, several drugs, including statins and anti-diabetic medications, are major agents used in therapy, which do little to treat the secondary symptoms and are associated with side effects (28, 29).

In addition, these therapeutic approaches focus on either glucotoxicity resulting from irregular and unmanageable blood glucose levels, or lipotoxicity caused by an imbalanced lipid profile. However, they do not tackle the immune system disturbances triggered by MeTS (1, 4). Advancing metabolic disruption, enhanced by aging-induced inflammatory disorders (inflammaging), leads to accumulation of lipids in the aging organs, coupled with immunosenescence (3) which further increases individuals’ risk of contracting infectious diseases. It is important to note that therapeutic strategies are used after the disease has already started and are not given as a preventative measure.

A continuous safe supplementation approach, which could serve as a prophylaxis before the onset of disease and an adjunct to existing treatments after disease onset, could be a holistic solution for which the A. pullulans’ novel strains-produced exopolysaccharide beta glucan-based biological response modifiers could be of potential use. The A. pullulans is a polyextremotolerant generalist black yeast belonging to the phylum Ascomycota, class Dothideomycete and order Dothideales having high levels of genetic recombination (30). The AFO-202 strain of this black yeast produced beta glucan, having been documented to alleviate glucotoxicity and enhance immunity (8-10), when combined with the N-163 strain produced beta glucan has significant balancing effects on the lipid profile, anti-inflammatory and anti-fibrotic effects with immunomodulation (12, 14-16), are further substantiated by the results of the present study.

In the present study in healthy Japanese men, AFO-202 has been shown to enhance the immune system, as observed from the increase in eosinophils and monocytes. A decrease in CRP observed in Group I (Figure 2C) with CRP being an acute phase reactant (31) and known to increase rapidly with the onset of cell injury and inflammation shows that the AFO-202 beta glucan has anti-inflammatory and immune enhancement potential. A significant increase in CD11b in the monocyte fraction was observed after 21 days of ingestion (Figure 4D). CD11b is expressed on monocytes, macrophages, dendritic cells, granulocytes, and NK cells and is an LPS receptor (32). It is associated with the bacteriophagocytic activity of phagocytes. The increase in CD11b in the monocyte fraction of Group II after 21 days of consumption compared with before can be considered as a manifestation of immune activation by N-163 beta glucan. There was no change in IgG or IgM levels in the blood throughout the study period in both test groups, but an increase in IgA levels along with the decrease in D-dimer by AFO-202 and of galectin, fibrinogen, and ferritin by the combination of AFO-202 and N-163 beta glucan (Group II) offers evidence in favour of the combined approach for addressing immune associated coagulopathy-associated risks in diseases such as COVID-19, in which a hyperactivated immune response affects the clotting pathway (13). The decrease in NLR with an increase in LCR and LeCR, all having been reported to be potential biomarkers of the underlying inflammation and hyperactivated immune response in COVID-19 (13), further substantiate the potent anti-inflammatory potential of these beta glucans. In terms of the secondary endpoints of glycaemic control and normalization of cholesterol levels, a longitudinal comparison showed a significant decrease in HbA1c and GA after 21 days of consumption in Group I and in T-Cho and LDL-Cho in Group II, suggesting that the synergistic intake of these beta glucans suppresses the increase in blood glucose level and lowers the cholesterol level, which could be useful in the context of metabolic disorders with underlying immune dysregulation, which again is a key factor associated with disease severity and mortality in COVID-19 (13), apart from application in management of MeTS and associated cardiac/cardiovascular abnormalities (33, 34). Though some of the parameters showed only a minor difference in values, the multi-system effects of these beta glucans are the main reason for recommending their use for health and wellness. The proposed mechanisms by which the AFO-202 and N-163 beta glucans produce beneficial effects include their recognition as pathogen-associated molecular patterns (PAMPs), through which they modulate the function of immune cells (35). The cytochrome P450 enzyme 7-hydroxylase (CYP7A1) catalyses the formation of primary bile acids and thereby regulates cholesterol synthesis and liver cholesterol excretion. Beta glucan regulates CYP7A1 and HMG-CoA, which in turn regulate cholesterol synthesis and its decomposition into bile acids. By regulating enzyme activity in the liver, the lipogenic effects of beta-glucans are elucidated (36, 37). Some of the metabolic effects of the beta glucans may also be mediated by the gut microbiota. As beta-glucans are resistant to digestion by gastric and pancreatic enzymes, they are fermented by the host’s microbiome in the colon and exert their effects in this manner. Viscosity-dependent health benefits of highly viscous fibres such as beta glucans also contribute to cholesterol-lowering and improved glycemic control. Through short-chain fatty acid (SCFA)-induced production of gut hormones, beta-glucan suppresses appetite and increases insulin sensitivity. Gastric emptying peptide and GLP-1 are hypothesised to be related to these alterations (38). Immune-mediated effects of glucan are primarily induced by pattern recognition receptors (PRRs). These include Dectin-1, CR3, TLRs, lactosylceramides, and scavenger receptors. Dectin-1 is the key beta glucan receptor. The recognition and binding of TLR and Dectin-1 control the immune response by modulating the release of pro- and anti-inflammatory cytokines (39). A so far unidentified beta-glucan receptor that induces an Akt/P13K-dependent anti-inflammatory response also contributes to the metabolic and immune effects [40]. Beta glucan is also a potent inducer of epigenetic and functional reprogramming of innate immune cells, a process known as «trained immunity» that improves the host’s response to infections (41). Beta glucan induces acquired immunity via histone modifications at gene promoters in human monocytes, which is accompanied by increased production of proinflammatory cytokines in response to a microbial challenge. The expansion of hematopoietic stem and progenitor cells in the bone marrow and the increase in myelopoiesis provide significant protection against infection. This protective signature of beta glucan is mediated by IL-1 signalling. Beta-glucans activate a variety of immune cells, including macrophages, neutrophils, monocytes, natural killer cells, and dendritic cells, by binding to immune receptors such as Dectin-1, complement receptor 3 (CR3), and TLR-2/6 (42). Beta glucans can also modulate the tumour microenvironment by bridging the innate and adaptive arms of the immune system and by altering the phenotype of immunosuppressive cells to make them immune-stimulatory, contributing to the effects against cancer (42).

Two critical areas of further research are essential for a holistic understanding of their benefits. One is the process of aging, against which all mechanisms must act, as aging is an inevitable phenomenon causing gradual loss of optimal functioning capability of the whole human body, from the cellular to organ level, and age-related cumulative pathogenesis, especially the immune system. The second essential component of research must be on the gut microbiota, also called the “second genome” (43), as their involvement and contribution to both metabolism balancing and immune modulation, besides neuronal implications for aging apoptosis, chronic micro-inflammation, and carcinogenesis (44), have been gaining strong evidence in the past decade. With the earlier studies of immune cell enhancement in young healthy volunteers (45) and elderly cancer patients (46) having been earlier proven as well with these beta glucans, a large population involvement to document the same would strengthen such findings. Also, the effects of the beta glucan supplementation on the food dynamics of the participants, post-intervention needs further research. As beta glucans are known for their pre-biotic effects, and their beneficial effects could be proven to correlate with the gut microbiota, we should be able to see an amalgamation of all these and their mechanisms of interaction to explain technically their various potentials in terms of prevention, prophylaxis, and as a therapeutic adjunct for both communicable and non-communicable diseases.

It an important limitation is that this study was performed in healthy volunteers as an exploratory study, which warrants further validation in translational models designed for specific diseases to confirm the efficacy in specific pathogenesis and in human clinical studies in target illnesses. Further, because these beta glucans have been in consumption for long, AFO-202 since 1996 and N-163 since 2018 with safety track record as a food supplement, apart from pre-clinical and clinical studies (7, 8, 10-12, 15), we did not include a control group, as the main objective was to compare the immunological benefits of AFO-202 beta glucan alone versus when it is combined with N-163 beta-glucan. Although the study sample was small, this study paves the way for future research on the effects of these safe nutritional supplements in prophylaxis and prevention of disease in high-risk individuals, such as those with MeTS, as well as in infectious and non-infectious immune-metabolic dysfunction-associated diseases such as COVID-19.

## Conclusion

In summary, this study has demonstrated that the AFO-202 beta glucan is capable of eliciting beneficial effects in balancing blood glucose, alleviating glucotoxicity with immune activation, while a combination of AFO-202 and N-163 beta glucans has significant anti-inflammatory and lipid profile regulating potential, thereby alleviating lipotoxicity. Therefore, these beta glucans, being safe to consume, may be incorporated as a new beneficial adjunct therapy for individuals with developing and established MeTS. Future studies in subjects with relevant illnesses are warranted to evaluate these beta glucans’ application as agents for prophylaxis and management of fibrosis-induced non-communicable diseases such as fatty liver disease and immune hyperactivation-related diseases, especially in communicable diseases such as COVID-19. Though the factors capable of determining a reduction in severity and mortality are different and their interactions are not yet fully understood, further elaborate research on evaluation of these beta glucans for their pre-biotic potentials in gut microbiota and related outcomes in managing chronic microinflammation, apoptotic mechanisms, and carcinogenesis could lead to evolution of knowledge and henceforth applications.

## Supplemental Materials

Additional material Supplementary PDF file supplied by authors.

**Figure:**
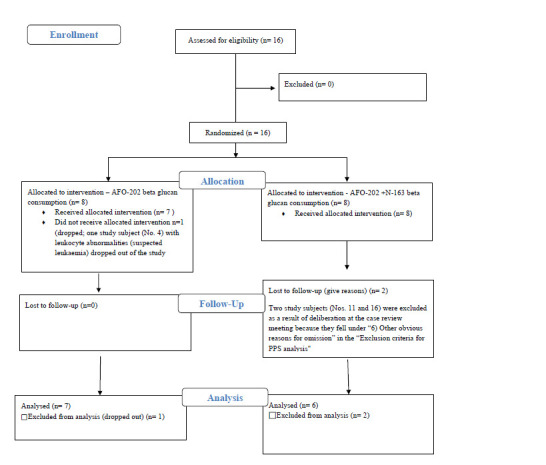
CONSORT Flow Diagram

## Data Availability

All data generated during the study are available in the manuscript itself.

## Author Declarations

Author Samuel Abraham is a shareholder in GN Corporation, Japan which in turn is a shareholder in the manufacturing company of novel beta glucans using different strains of Aureobasidium pullulans. The other authors don’t report any potential conflict of interests.
