# Supplementary material for: Beta 1,3-1,6 Glucans Produced by Two Novel Strains of Aureobasidium Pullulans Exert Immune and Metabolic Beneficial Effects in Healthy Middle-aged Japanese Men: Results of an Exploratory Randomized Control Study
Source: JAR Life. 2023 Jul 28;12:61–71. doi: 10.14283/jarlife.2023.11 (PMC10457473; doi:10.14283/jarlife.2023.11)
Supplement: Additional material — Supplementary PDF file supplied by authors. [file jarlife-12-011-S1.doc]

**CONSORT Flow Diagram**

**Allocation**

**Analysis**

**Follow-Up**

**Enrollment**

Assessed for eligibility (n= 16)

Excluded (n= 0)

Analysed (n= 7)
 Excluded from analysis (dropped out) (n= 1)

Lost to follow-up (n=0)

Allocated to intervention – AFO-202 beta glucan consumption (n= 8)

- Received allocated intervention (n= 7 )
- Did not receive allocated intervention n=1 (dropped; one study subject (No. 4) with leukocyte abnormalities (suspected leukaemia) dropped out of the study
- out after randomization) (n=1)

Lost to follow-up (give reasons) (n= 2)

Two study subjects (Nos. 11 and 16) were excluded as a result of deliberation at the case review meeting because they fell under “6) Other obvious reasons for omission” in the “Exclusion criteria for PPS analysis"

Allocated to intervention - AFO-202 +N-163 beta glucan consumption (n= 8)

- Received allocated intervention (n= 8)

Analysed (n= 6)
 Excluded from analysis (n= 2)

Randomized (n = 16)
